# Evidence for X-Chromosomal Schizophrenia Associated with microRNA Alterations

**DOI:** 10.1371/journal.pone.0006121

**Published:** 2009-07-01

**Authors:** Jinong Feng, Guihua Sun, Jin Yan, Katie Noltner, Wenyan Li, Carolyn H. Buzin, Jeff Longmate, Leonard L. Heston, John Rossi, Steve S. Sommer

**Affiliations:** 1 Division of Molecular Genetics, City of Hope National Medical Center, Duarte, California, United States of America; 2 Department of Molecular Biology, City of Hope National Medical Center, Duarte, California, United States of America; 3 Graduate School, City of Hope National Medical Center, Duarte, California, United States of America; 4 Division of Information Sciences, City of Hope National Medical Center, Duarte, California, United States of America; 5 Department of Psychiatry, University of Washington, Seattle, Washington, United States of America; 6 MEDomics, Azusa, California, United States of America; University of Wuerzburg, Germany

## Abstract

**Background:**

Schizophrenia is a severe disabling brain disease affecting about 1% of the population. Individual microRNAs (miRNAs) affect moderate downregulation of gene expression. In addition, components required for miRNA processing and/or function have also been implicated in X-linked mental retardation, neurological and neoplastic diseases, pointing to the wide ranging involvement of miRNAs in disease.

**Methods and Findings:**

To explore the role of miRNAs in schizophrenia, 59 microRNA genes on the X-chromosome were amplified and sequenced in males with (193) and without (191) schizophrenia spectrum disorders to test the hypothesis that ultra-rare mutations in microRNA collectively contribute to the risk of schizophrenia. Here we provide the first association of microRNA gene dysfunction with schizophrenia. Eight ultra-rare variants in the precursor or mature miRNA were identified in eight distinct miRNA genes in 4% of analyzed males with schizophrenia. One ultra-rare variant was identified in a control sample (with a history of depression) (8/193 versus 1/191, p = 0.02 by one-sided Fisher's exact test, odds ratio = 8.2). These variants were not found in an additional 7,197 control X-chromosomes.

**Conclusions:**

Functional analyses of ectopically expressed copies of the variant miRNA precursors demonstrate loss of function, gain of function or altered expression levels. While confirmation is required, this study suggests that microRNA mutations can contribute to schizophrenia.

## Introduction

Schizophrenia typically presents in early adulthood or late adolescence. Men have an earlier age of onset than women, and also tend to experience a more serious form of the illness with more negative symptoms, poorer chances of a full recovery, and a generally worse outcome [1]. Systematic reviews indicate that schizophrenia seems more common in men than in women (risk ratio of above 1.4∶1).

MicroRNAs (miRNAs) are a large family of small, non-coding RNAs that negatively regulate gene expression at the post-transcriptional level [2]–[4]. In animals, miRNAs bind to complementary sites in target mRNAs, generally at 3′ untranslated regions (UTRs), to create imperfectly paired miRNA/mRNA heteroduplexes that inhibit translation or increase degradation of target mRNAs.

MiRNA genes are scattered among all the chromosomes in humans, except for the Y- chromosome. Most miRNAs are Pol II transcripts with a 5′ Cap and 3′ Poly(A) tail termed primary miRNA (pri-miRNA) [5]. The pri-miRNAs are processed to hair-pin like structures termed precursor miRNA (pre-miRNA). The terminal loop of the pre-miRNAs are cleaved to generate miRNA/miRNA* partial duplex structures. One of the strands is then preferentially loaded in miRNA induced silence complex (RISC). Mature miRNAs often target 3′UTRs and reduce protein expression. Translational suppression and mRNA degradation, modes by which mammalian miRNAs regulate gene expression, do not require complete complementarity between the miRNA and target. Watson-Crick base pairing between seven consecutive nucleotides in the target mRNA's and nucleotides 2–8 (the “seed sequence”) at the miRNA's 5′ end is generally sufficient. For the majority of miRNA/target combinations, the seed sequence complementarity is a pre-requisite, although there are some exceptions which complicate target site predictions. For most miRNA/target combinations, a single nucleotide change in the seed sequence or a mutation in the miRNA or precursor affecting the Drosha/DGCR8 or Dicer/TRBP processing step can result in altered function or creation of a novel miRNA [6]–[9].

It is estimated that approximately one-third of human protein coding genes are post-transcriptionally controlled by miRNAs (reviewed in [10]). Many microRNAs are conserved in sequence and function between distantly related organisms [11].

miRNAs regulate various biological functions including developmental timing, cell proliferation, neuronal cell fate, apoptosis (reviewed in [12], [13]), neuronal gene expression [14], brain morphogenesis [15], muscle differentiation [16] , and stem cell division [17]. It is speculated that condition-specific, time-specific, and individual-specific levels of gene expression may be due to the interactions of different miRNAs accounting for more accurate genetic expression of various traits [18]. The large number of miRNA genes, the diverse expression patterns and the abundance of potential miRNA targets suggest that miRNAs may be a significant but unrecognized source of human genetic disease, including neuropsychiatric disorders. A sequence variant in the binding site for the miRNA miR-189 in the SLITRK1 (MIM # 609678) mRNA has been shown to be associated with Tourette syndrome [19]. However, two recent studies of ∼800 and ∼2300 Tourette syndrome patients and family members found non-transmission of the variant in two families in each study from parent to affected child [20], [21] the former study suggested that the variant may be a rare Ashkenazi Jewish population variant. In addition, components required for miRNA processing and/or function have also been implicated in fragile X mental retardation [22], chromosome 22q11 deletion syndrome (DiGeorge syndrome) [23], [24] and cancer [25], pointing to the wide ranging involvement of miRNAs in disease.

To explore the possibility that high risk structural variants in the microRNA genes could predispose to schizophrenia, we chose the X-chromosomal miRNA genes available at the time of the study. 59 microRNA genes on the X-chromosome were sequenced in 193 unrelated male patients with schizophrenia and 191 male controls. The one tested hypothesis is that ultra-rare variants in the X-linked microRNAs predispose to schizophrenia, consistent with reduced fertility in schizophrenia and consequent elimination of high risk X-linked mutations within a few generations [26]. This analysis should be distinguished from linkage disequilibrium/Hap Map analyses in which thousands of hypotheses are typically being tested.

## Materials and Methods

### Samples

All 193 male Caucasian schizophrenic patients met criteria for the disease as defined by the Diagnostic and Statistical Manual, Fourth Edition, Revised (DSM-IV-R), as described previously [27]. In brief, schizophrenic patients were ascertained from a variety of inpatient and outpatient centers in south Minnesota. These included two state hospitals, two tertiary care hospitals, and two outpatient mental health centers. Institutional review board approval for studies with human subjects was granted by each participating institution, including the Institutional Review Board of the City of Hope, IRB#99065, and all participants signed informed consent statements. All cases were diagnosed by a research psychiatrist based on Diagnostic and Statistical Manual, Fourth Edition, Revised (DSM-III-R) criteria. Strict fulfillment of the diagnostic criteria was assessed primarily through the review of medical records. The southern Minnesota region has a limited number of psychiatric and medical care sources and multiple medical records covering the course of entire illness were often available. The male controls were Midwest Caucasians with no known history of diagnosed psychiatric illness.

### PCR Amplification and sequencing

The genomic sequence and adjacent flanking sequences of the precursors of 59 X-linked microRNA genes (miRBase 10.1, http://microrna.sanger.ac.uk/sequences/) were amplified and sequenced with the ABI model 3730 sequencer. The miRNA accession numbers and primer sequences are listed in Table S1. The nucleotide alterations were analyzed with Sequencher software™ (Gene Codes, Ann Arbor, MI). Mutations were confirmed by reamplifying from genomic DNA and sequencing in the opposite direction.

**Table 1:** Ultra-rare miRNA cohort-specific variants.

ID#	Disease	miRNA	Variant in mature miRNA	Variant in precursor	Gene pool
S358	Schizophrenia	let-7f-2	11 G>A		0/7,197
S418	Schizophrenia	miR-18b		32 A>G	0/7,197
S590	Schizophrenia	miR-505		8 C>T	0/7,197
S356	Schizophrenia	miR-502		13 C>G	1/7,197^a^
S014	Schizophrenia	miR-188	7 C>T (188-3p)		0/7,197
MC179	Psychosis	miR-325	8 G>A (325-3p)^b^		0/7,197
S711	Schizophrenia	miR-660	15 C>T		0/7,197
S596	Schizophrenia	miR-509-3	13 C>T (509-3p)		0/7,197
MC149	Control^c^	miR-510	4 T>C (510-3p)		0/7,197

a One individual in the initial gene pool sample had the variant, but was excluded from the sample when a history of depression was found on review of the medical records. The extent of the depression requires further clarification.

b The exact sequence for the mature 3p is not known; the numbering is based on the predicted sequence.

c Thirty-four years of medical history until death included depression, gout, closed angle glaucoma, tricuspid and aortic insufficiency, congestive heart failure, and mitral aortic prosthesis.

### Gene pool analysis

A gene pool analysis [28] was performed to identify the private mutations from high risk X-chromosomal variants that predispose to schizophrenia, which is associated with reduction in offspring. The high risk alleles are ultra-rare, i.e. not expected even once in the gene pool analysis. Less than 1 per 250,000 controls (1 per 500,000 control alleles) is expected to have the mutation at random if: i) 1% or less of the population has schizophrenia; ii) 2% of these patients have high risk X-linked miRNA mutations expected to impart lower reproductive fitness (especially before the development of effective drug therapy); and iii) the eight ultra-rare variants found in schizophrenia constitute less than 2% percent of the total miRNA mutations. Thus, such variants are not expected in 7,197 control X-chromosomal alleles.

Control individuals (10,000 autosomal alleles, 7197 X chromosome alleles and 2803 Y chromosome alleles) were ascertained from a Midwest population-based sample from Minnesota and a more ethnically and geographically diverse sample of hemophilia families. The total ethnicity distribution is as follows: 86.8% European Caucasians, 4.3% Hispanics, 2.2% Asians, 2% Blacks, 2% Mestiza Columbian, 0.8% American-Indians and 1.9% of unknown ethnicity. The average age of the individuals included in the gene pool is 44 years, so most of these individuals would have manifested schizophrenia if that were their destiny. To our knowledge there is no relationship between hemophilia and schizophrenia; none of the patients with hemophilia are known to have schizophrenia.

The concentration of individual DNA samples was estimated by both UV spectrophotometry and agarose gel electrophoresis with diluted quantitation standards. Samples were diluted to 200 ng/µl and combined into pools of 10, 30 and 100 samples. The concentration of each sample per µl in the pool is 20 ng, 6.7 ng and 2 ng, respectively.

Allele-specific amplification assays were developed for each case or control cohort-specific variant [29]–[31]. The specificity and selectivity of each assay were determined utilizing negative and positive controls spiked within gene pool samples. As previously described, the sensitivity of the allele-specific PCR assay must be at least four-fold greater than the number of pooled alleles in the sample, i.e. for autosomal genes, a 30-in-one sample pool contains 60 alleles. Thus, spikes within 30-in-one pools must be able to detect signal when the allele is present at one part in 240. For pools of 100 in 1, an allele must be detected with a sensitivity of at least one part in 800. Allele-specific PCR is optimized in the manner previously shown to produce specificity for 69 consecutive assays [30]. To facilitate timely optimization, eight oligonucleotides are routinely generated: i) Two allele-specific downstream oligonucleotides utilizing the 3′ nucleotide for allele specificity and differing in length (Wallace temperature [4°×(G+C)+2°×(A+T)] of about 46/48 or 50/52 degrees, respectively) were paired with either of two generic upstream oligonucleotides to form amplicons of 150–250 bp and ii) two upstream allele-specific oligonucleotides utilizing the 3′ nucleotide for allele specificity (Wallace temperatures 46/48 or 50/52 degrees) are generated and paired with either of two generic downstream oligonucleotides to form amplicons of about 150–250 bp. After an initial optimization for oligonucleotide concentration and magnesium concentration, an allele-specific reaction with the required sensitivity is used for the gene pool assay. If none achieve the required sensitivity, further optimization is performed as previously described [30]. The assay conditions for each of the ultra-rare assays are as described (Table S2).

### Cell lines and plasmids

HEK293, Hela, NIH-3T3 cells were purchased from ATCC and maintained in high glucose (4.5 g/l) DMEM supplemented with 2 mM glutamine, 10% FBS, and 2 mM Penicillin/Streptomycin. Transfections to HEK293, NIH-3T3 and Hela cells were performed with Lipofectamine 2000 (Invitrogen) in duplicate in 24-well plate formats when cells are at 70–80% confluency.

### Cell based miRNA processing test

Primary miRNA (pri-miRNA) expression plasmids and reporters bearing either fully complementary or seed sequence complements to the miRNAs were co-transfected into HEK293 cells. Dual-reporters (expressing both Firefly and Renilla luciferase) carrying the miRNA fully complementary sequences (si reporter) in the 3′ UTR of the Renilla transcript were used to validate the ability of cloned pri-miRNA expression plasmids to produce functional, mature miRNAs. Dual-reporters carrying the partially complementary sequence (mi reporter: mis-matched at position 11 to 13 and the last two nts in miRNA/mRNA duplex) of a miRNA in the 3′UTR of Renilla were used to measure the strength of translational repression from the corresponding miRNA.

In order to express the pri-miRNAs, we retrieved the stem-loop sequences from miRBase 10. The stem-loop sequence plus flanking sequences extending over 100 bases in both directions, was PCR amplified from genomic DNA. A miRNA expression vector was constructed by first cloning the human Pol II U1 promoter upstream of a multiple cloning site in the Bluescript SK plasmid to create SK-U1. Secondly, the U1 transcriptional termination sequence was cloned downstream of the MCS of SK-U1 to create the fU1-miR miRNA expression vector. The primary miRNA was cloned into the Xho I and BamH1 sites of fU1-miR. miRNA variants were cloned in the same manner as the wild type miRNAs from patient DNA when available. If samples were no longer available, the QuikChange II site-directed mutagenesis kit was used to create mutants within the wildtype expression constructs. All clones were sequenced to confirm the normal miRNA and mutant forms.

For si reporters, all miRNAs and their homologous mutant target sequences were designed as fully complementary to the mature miRNA sequence. The oligonucleotides for the two strands were inserted into the psiCHECK Xho I/Spe I or XhoI/Not I digested reporter 3′ UTR of the Renilla luciferase gene. All target clones were verified by sequencing. For miRNA reporters, all the inserted sequences in the reporter 3′ UTR of the Renilla luciferase gene were designed with bulges at positions 11 to 13 and were unpaired for two nucleotides at the 3′ end of the miRNA.

About 1×10ˆ5 HEK293 cells per well in 500 microliters of growth media were plated in 24-well plates one day prior to transfection. The cells were at 70–80% confluency at the time of transfection. Each well was transfected with 5 ng reporter, 100 ng miRNA expression constructs (1∶20 ratio, 1∶5 ratio was used if the knockdown of the si target was >95%: 25 ng of miRNA expression plasmid and 75 ng stuffer Blue-script SK) and 1 ul Lipofectomine 2000. Forty eight hours post transfection, the cells were lysed with 100 µl Passive lysis buffer (Promega) and luciferase levels were analyzed from 20 µl lysates using the Dual Luciferase reporter assay (50 µl of each substrate reagent, Promega) on a Veritas Microplate Luminometer (Turner Biosystems). Changes in expression of Renilla luciferase (target) were calculated relative to Firefly luciferase (internal control) and normalized to the miRNA expression vector control fU1-miR.

Point mutations were created with the QuikChange site-directed mutagenesis kit II (Stratagene) following the protocol included in the kit. Mutations were confirmed by sequencing.

### Northern blot

Two different transfections were performed in HEK 293 cells to detect processing of expressed pri-miRNA in vivo. One transfection contained pri-miRNA expression constructs alone, while the other was co-transfected with 27-mer synthesized siRNA duplex. Northern blots were performed with RNAs from both transfections. U2 was used as the RNA loading control and co-transfected siRNA-1 that target HIV Tat/Rev was used as the transfection control. Northern blots were performed according to a previous publication [32]. Briefly, 20 µg total RNA was loaded on a 12.5% SDS-PAGE gel. Gels were transferred to a Hybond-N+ (Amersham Pharmacia biotech, positive charged) membrane. DNA probes were used for all Northern blots. Hybridizations were carried out in PerfectHyb™ Plus hybridization buffer (Sigma) for 16 hours (Table S3).

#### Statistical Considerations

The hypothesis considered here is that ultra-rare, essentially private, mutations in the X-linked miRNA are collectively associated with schizophrenia. This hypothesis, or any hypothesis about a collection of mutations, admits the possibility of including mutations with a wide range of penetrance. Because the phenotype has likely been subject to a substantial selective pressure, the set of rare alleles is likely to include more genes of high penetrance, and fewer neutral genes, compared to more frequent alleles, all else being equally likely. A total of 384 X-linked alleles of the miRNA genes were sequenced in order to discover multiple rare alleles. Mutation discovery was necessary in controls as well as cases, in order to distinguish a substantial risk attributable to rare alleles collectively from a high frequency of neutral mutations. Restricting attention to mutations detected only once in the sequencing effort would provide a measure of rarity, but an additional requirement that the variants be absent from a pool of 7,197 X chromosomal alleles served to further exclude low-frequency neutral alleles in favor of essentially private mutations. Because variants were ascertained by sequencing, the frequency with which the variants were detected in the pooled alleles cannot serve as a reference for statistical comparison. After defining ultra-rare variants to be those detected only in either sequenced cases or sequenced controls and not in the gene pool control alleles interrogated, the primary analysis is simply a comparison of the number of ultra-rare alleles among 193 cases to the number among 191 controls, using Fisher's exact test (StatXact, CYTEL, Cambridge, MA). We regard an unadjusted, one-sided significance probability (p-value) as relevant to the testing of this single hypothesis. The confidence interval of the estimate for attributable risk is a binomial confidence interval calculated by the “Blyth-Still-Casella” method in StatXact.

## Results

Eight variants in the precursor or mature miRNA were identified in eight distinct miRNA genes in males with schizophrenia and one ultra-rare variant was identified in a control sample (with a history of depression) (8/193 versus 1/191, p = 0.019, Fisher's exact test) (Table 1). These variants are ultra-rare, as determined by gene pool analyses of an additional 7,197 control X-chromosomes (Materials and Methods). Each ultra-rare variant was present in a different individual.

Five cohort-specific variants, one in a patient and four in controls, were found in the gene pool analyses at a frequency greater than 0.02% (p = 0.21, Fisher's exact test) (Table 2). Five common variants in miRNA precursors were found in both patients and controls with similar frequencies (Table S4).

**Table 2:** miRNA cohort-specific sequence variants found in the gene pool analyses.

ID#	Disease	miRNA	Variant in mature miRNA	Variant in precursor	Gene pool
S464	Schizophrenia	miR-509-3	22 G>A (509-3-5p)		2/7,197
MC527	Control	miR-509-3	19 C>G (509-3-5p)		10/4,962
MC333	Control	miR-421		73 G>A	16/4,962
MC40	Control	miR-934	1 T>G		4/7,197
MC93	Control	miR-450-2	4 T>C		8/4,962

**Figure 1:**
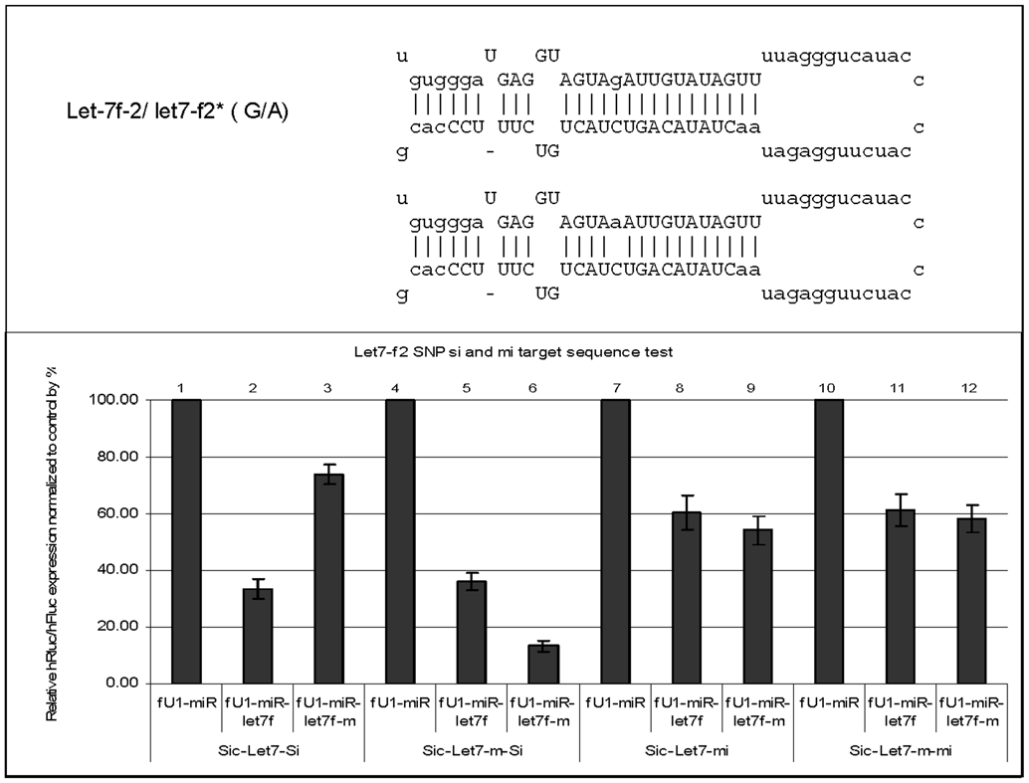
Function test of let-7f-2-G/A. A single base substitution G>A was identified in the mature miRNA of let-7f-2 at position 11. To examine the possible functional consequences of this mutation, the wild type and mutant variants were tested against its corresponding ‘si’ and ‘mi’ target sequence. The results obtained with these analyses demonstrate that the mutant sequence can downregulate its own fully complementary ‘si’ sequence (bar #6), but its knockdown of the let-7f ‘si’ sequence was dramatically reduced (bar #3). On the other hand, the let-7f knockdown of the mutant ‘si-target’ remained unperturbed (bar #5). These results demonstrate that the mutant produces a stronger siRNA phenotype than the wild type miRNA with the cognate complementary targets. On the other hand, the variant elicits a weaker miRNA phenotype than the wild type. Sic-[target]-Si and Sic-[target]-Mi: Dual reporters containing the miRNA target sequences (Si, fully complementary; Mi, partially complementary) in the 3′UTR of the Renilla luciferase gene (for details, see Materials and Methods). fU1-miR-[miRNA] and fU1-miR-[miRNA]-m: miRNA expression vectors containing the primary sequence of a specific miRNA gene (wild type and mutant, respectively) (for details, see Materials and Methods). fU1-miR: Expression vector alone without the miRNA gene inserted.

**Figure 2:**
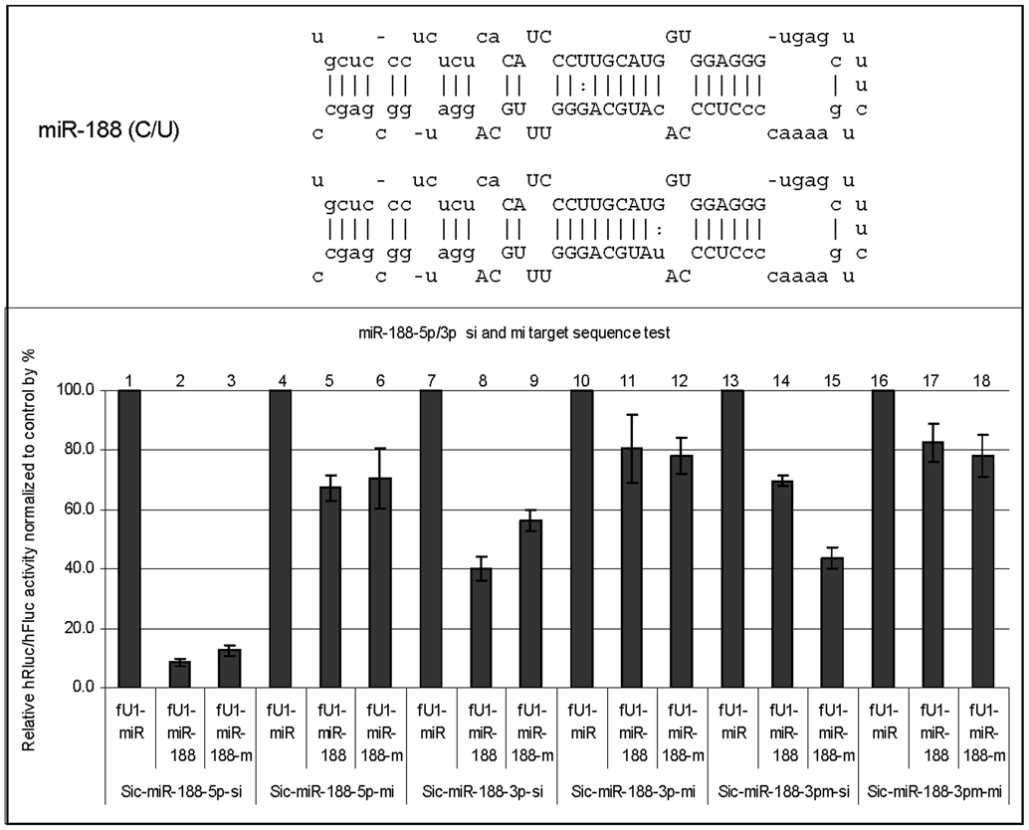
Function test of miR-188-5p/3p. Variant miR-188-5p/3p-m has a ‘C’ to ‘T’ (U) transition at the 7th nt of the mature miR-188-3p within the seed sequence. This variant results in a change of G∶C to G∶U pairing in the seed sequence. In our assay system, the effect of the variant is not dramatic. Nevertheless, this variant will create a seed sequence where this position can pair with an A, thus potentially affecting the expression of new target sequences with a matched seed sequence. Sic-[target]-Si and Sic-[target]-Mi: Dual reporters containing the miRNA target sequences (Si, fully complementary; Mi, partially complementary) in the 3′UTR of the Renilla luciferase gene (for details, see Materials and Methods). fU1-miR-[miRNA] and fU1-miR-[miRNA]-m: miRNA expression vectors containing the primary sequence of a specific miRNA gene (wild type and mutant, respectively) (for details, see Materials and Methods). fU1-miR: Expression vector alone without the miRNA gene inserted.

To assess the functional consequences of the point mutations, pri-miRNAs and mutant versions of each miRNA were co-transfected with their corresponding siRNA (si) and miRNA (mi) targets (see Materials and Methods). Three or more transfections were performed, with duplicates in each transfection. Five of the variants identified in patients, as well as one identified in a control sample (with depression), contained a point mutation in the mature coding region (miRNA let-7f-2, miR-188, miR-325, miR-660, miR-509-3, and miR510) (Figures 1, 2 and Figs. S1, S2, S3; S5). These novel, ultra-rare variants in the mature miRNAs were predicted to have altered target specificity and perhaps new endogenous targets. The results of the *in vitro* functional assays for the nine variants are described below. (In all pictures: the mature sequences in the stem-loop structure are in uppercase except SNPs in mature sequence are in lower case; sequences outside the mature sequences are in lower case except SNPs outside the mature sequences are in uppercase.)

### Let-7f-2: 11 G>A

This variant was identified in the mature miRNA at position 11 (Fig. 1). In the functional assays, the wild type let-7f-2 knocks down expression of its complementary ‘si’-mutant target; the mutant miRNA is much less effective in knockdown of the wild type target. On the other hand, the mutant miRNA strongly down-regulates its complementary ‘si’- mutant target, while the wild type miRNA has about the same knockdown of the mutant and wild type targets.

### miR-188: 7 C>T (3p)

This variant occurs within the seed sequence at the 7th nucleotide of the mature miR-188-3p (Fig. 2). In our assay system, the effect of the 5p variant is not dramatic. The 3p variant is less effective than the wild type 3p in suppressing expression of the ‘si’ target, although there is no difference with the ‘mi’ target. With the mutant ‘si’ target, the knock-down by the variant miRNA is enhanced compared to the wild type miRNA. In addition to the functional tests shown herein, this variant in the seed sequence could potentially affect the expression of new target sequences with a sequence matched to the variant.

### miR-18b/18b*: 32 A>G

**Figure 3:**
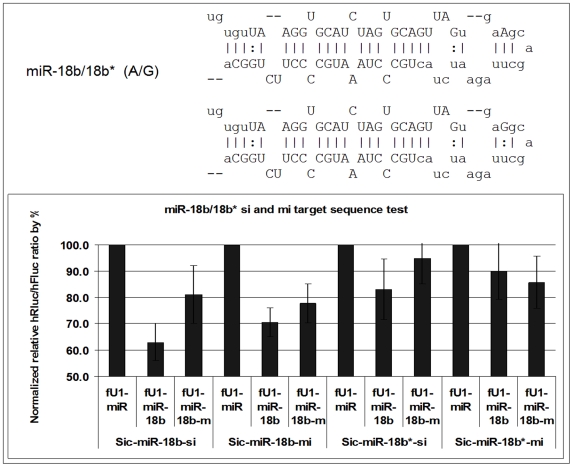
Function test of miR-18b-A/G. Variant miR-18b/18b*-m has an ‘A’ to ‘G’ mutation at the 4th nt following the last base of the mature sequence, which is also in the predicted terminal loop structure. This sequence difference may affect processing and/or stability as there is a reduction in the level of target knockdown activity when compared to wild type in the ‘si-target’ (bar #2 vs #3) and the ‘mi-target’ assays (bar #5 vs #6). In contrast, the function of the miR-18* strand does not appear to be affected by this mutation (bar#8 vs 9 and Bar #11 vs 12). Sic-[target]-Si and Sic-[target]-Mi: Dual reporters containing the miRNA target sequences (Si, fully complementary; Mi, partially complementary) in the 3′UTR of the Renilla luciferase gene (for details, see Materials and Methods). fU1-miR-[miRNA] and fU1-miR-[miRNA]-m: miRNA expression vectors containing the primary sequence of a specific miRNA gene (wild type and mutant, respectively) (for details, see Materials and Methods). fU1-miR: Expression vector alone without the miRNA gene inserted.

This variant occurs at the 4th nt following the last base of the mature sequence in the terminal loop close to the Dicer/TRBP processing site (Fig. 3). The mutant miRNA has decreased activity when compared to wild type miRNA in the ‘si target’ and the ‘mi target’ assays. The 18b* strand also has reduced activity compared to wild type in the mutant-‘si target’ assay, although there is essentially no difference between wild type and mutant in the mutant-‘mi target’ assay.

### miR-502: 13 C>G

**Figure 4:**
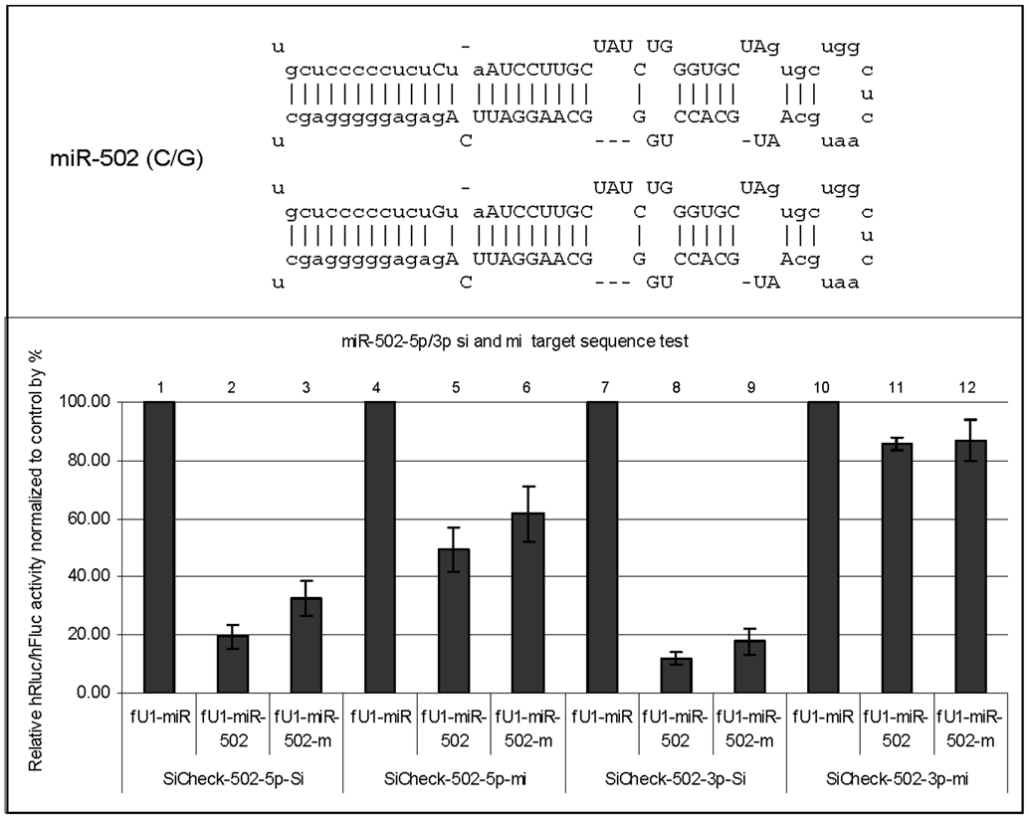
Function test of miR-502-C/G. Variant miR-502-5p/3p-m has a ‘C’ to ‘G’ transversion at the 3^rd^ nt upstream of the mature miR-502-5p sequence. This mutation will produce a bulge which changes the structure of the stem of the precursor miRNA. Most likely, this structural change will affect the site of Drosha cleavage in producing pre-miR-502; therefore, both the 5p and 3p products should be affected. Reduced target knockdowns were observed in transfection assays (bar #2 vs 3, # 5 vs 6 and # 8 vs 9). Sic-[target]-Si and Sic-[target]-Mi: Dual reporters containing the miRNA target sequences (Si, fully complementary; Mi, partially complementary) in the 3′UTR of the Renilla luciferase gene (for details, see Materials and Methods). fU1-miR-[miRNA] and fU1-miR-[miRNA]-m: miRNA expression vectors containing the primary sequence of a specific miRNA gene (wild type and mutant, respectively) (for details, see Materials and Methods). fU1-miR: Expression vector alone without the miRNA gene inserted.

**Figure 5:**
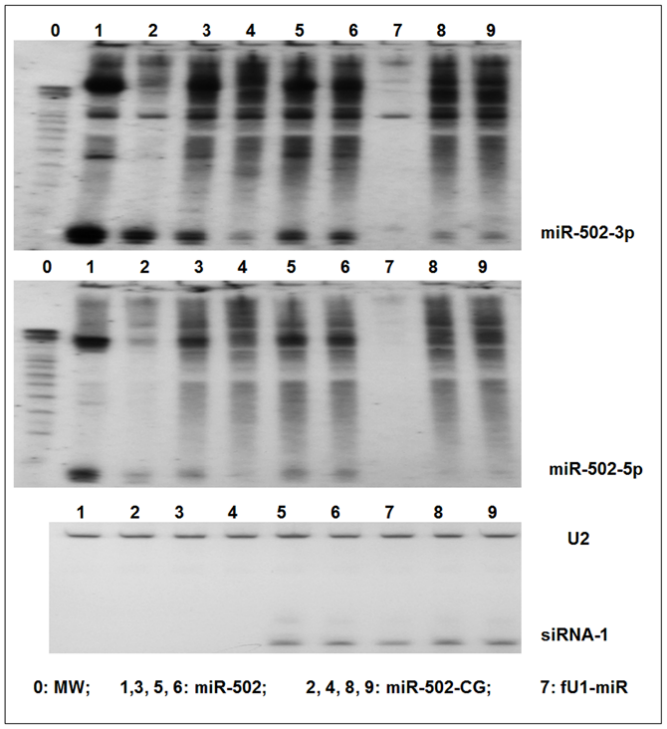
Northern blot results of miR-502-C/G. The impaired functional activity of the variant miR-502-C/G was supported by Northern blot analyses, as the production of pre-miR-502 and mature 502-5p/3p were both reduced. Top is the result of the blot that was hybridized with miR-502 3p probe; middle is the result of the blot that was hybridized with the 5p probe; bottom is the result of the blot that was hybridized with U2 snoRNA probe and spike-in siRNA probe. U2 was used as RNA sample loading control. SiRNA-1 that targets HIV Tat/Rev was used as a transfection control. Lanes 1, 3, 5, and 6 are miR-502; Lanes 2, 4, 8, and 9 are transfected with the variant. Lane 7 is the expression vector alone: fU1-miR.

This variant is of interest, as it occurs in the precursor miRNA at 3 nucleotides before the Drosha/DGCR-8 processing site (Figure 4). The mutation should produce a bulge, which will change the structure of the stem of the precursor miRNA, and will likely affect the site of Drosha cleavage in producing pre-miR-502. As expected, both the 5p and 3p products are affected: 5p slightly reduces the ‘si’ and ‘mi’ functions, while 3p slightly reduces the ‘si’ function. A Northern blot also shows a decreased level of both the 5p and 3p mature miRNA strands (Figure 5).

### miR-510: 4 T>C (3p)

This variant, which was detected in a control sample, occurs in the seed sequence at the 4^th^ nucleotide of the predicted miR-510-3p (Fig. S1). The mutant miRNA (both the 5p and the 3p strands) has much less activity than the wild type in the knock-down of ‘si’ expression; the 5p mutant strand (but not the 3p) also is less active in suppressing ‘mi’ expression. This mutation most likely affects the structure of the pre-miR-510, as it affects the function of mature miR-510 on both strands. The Northern blot shows that both the 5p and 3p products are reduced (Fig. S1a and S1b).

### miR-660: 15 C>T

This variant occurs at the 15th position of the mature miR-660-5p, but it is not in the seed sequence (Fig. S2). The functional data show that both the variant and wild type miRNAs are equally strong suppressors of both the 5p ‘si’ and ‘mi’ target sequences. The mutant miRNA has an enhanced 3p ‘si’ function compared to the wild type miRNA, which has no effect on 3p ‘si’ function. In addition to the functional tests herein, the base change could affect the natural targeting functions of the miRNA since this position affects the 3′end base pairing of miRNA/mRNA.

### mir-325: 8 G>A (3p)

This variant occurs at the 8^th^ position of the mature miR-325-3p strand (Fig. S3). The functional assays are indeterminate: neither the wild type nor mutant miRNA generated knockdowns of the wild type ‘si’ or ‘mi’ targets. At this time, there is no information concerning whether or not miR-325* (3p) is functional. This miRNA was originally cloned in a murine system and therefore there is no data demonstrating that the human homologue can be expressed and processed in HEK293 cells. We also analyzed processing of this miRNA in Hela and NIH 3T3 cells, and in both cases were unable to detect knockdown of the complementary target. It is also possible that this miRNA may be processed through an alternative miRNA pathway [33], [34].

### miR-505: 8 C>T

This variant occurs at the 8^th^ nucleotide of the precursor miRNA and is distal to both the Drosha and Dicer cleavage sites (Fig. S4). Functional testing of the mutant shows essentially no difference between the mutant and wild type miRNAs for both the ‘si’ and ‘mi’ targets.

### miR-509-3: 13 C>T (3p)

This variant is in the mature miR-509-3p at position 13, but it is not in the seed sequence (Fig. S5). There is essentially no difference in the mutant and wild type miRNAs with respect to ‘si’ or ‘mi’ function and none would be predicted from the position of the alteration. Since the mutation is not in the seed sequence, it is unknown whether the target specificity will be changed. It is possible that the base change could affect the regulation of endogenous targets, as the 13^th^ position is important for 3′ end base pairing.

## Discussion

We chose to examine hemizygous miRNA on the X-chromosome in males so that the full phenotypic consequences of a variant could be assessed without the possible mitigating effects of a wild-type allele.

The assessment of statistical significance is based on a single test of the collective effect of all microRNAs on the X-chromosome. This is similar to the strategy of Walsh et al. who tested the collective effect of individually rare copy number variants on the risk of schizophrenia [35]. The estimated odds ratio of 8.2, combined with the 1% lifetime incidence of schizophrenia, implies an estimated risk of 7.9% among individuals harboring a rare mutation, i.e. an estimated relative risk (RR) of 7.9 (95% CI 1.34–300). This is consistent with the finding that most of the rare mutations detected had a likely impact on regulatory function. The estimated overall attributable risk of the X-linked miRNAs is 0.036. Functional analyses indicated that some of these variants display altered regulatory function consistent with dominant inheritance. If similar risk levels apply to the 90% of microRNAs on autosomes, with half of the variants having dominant effects, then the risk attributable to rare microRNA mutations would be approximately 20%. This is, of course, a very rough estimate, but the data implicate microRNA mutations in schizophrenia, and suggest that their contribution to disease etiology may be substantial.

The altered functions or defects in processing of the pre-miRNAs in the mutant alleles detected in our analyses suggest that these microRNAs may contribute to the development of schizophrenia. It is important to note that each microRNA can potentially regulate dozens, and perhaps even hundreds of different transcripts during development, so even subtle defects in activity can have profound effects on development of the nervous system.

Identified targets of the miRNAs in which ultra-rare variants were found are listed in Table S5. Such targets include neuregulin 1 (NGR1), Disrupted in schizophrenia 1 (DISC1) and Regulator of G-protein signaling 4 (RGS4). These genes have been frequently studied and are commonly considered the best candidate genes for schizophrenia [36]. Defects in miRNAs altering the interactions between miRNAs and their mRNA targets may contribute to schizophrenia.

Six of the nine ultra-rare variants occur in the miRNA mature sequence (Table 1): Since miRNAs rely on base-pairing for their function, especially the ‘seed’ sequence base pairing, these variants are expected to have different regulatory specificities. All variants that occur in the mature miRNA sequence are expected to have altered function because the target spectra are changed. However only two of the variants in the mature miRNA sequences exhibit a strong alteration in the *in vitro* functional assays that measure a subset of miRNA function; the *in vitro* function in two other variants with alterations in the pre-miRNA is also affected.

In summary, these studies represent the first statistically significant association between microRNA mutant alleles and schizophrenia. The absence of variants in 7,197 and additional control X-chromosomes and abnormal functional studies support an etiological link. Future studies will be required to confirm the association and to determine whether autosomal miRNAs are also implicated in disease. The dominant phenotype suggested by some of the functional analyses herein is consistent with autosomal dominant as well as autosomal recessive inheritance of predisposition to schizophrenia. The extrapolation from our data to possibly 20% of schizophrenia resulting from miRNA mutations involves multiple caveats, including: i) replication of the X-chromosomal association and behavioral studies of mouse knock-ins are important to verify both the association and the attributable risk for X-chromosomal miRNA mutations; ii) there are major uncertainties in the estimate of miRNA causing schizophrenia by a dominant mechanism and iii) there are no data at present on the frequency of autosomal recessive miRNA variants that predispose to schizophrenia.

At least two important clinical implications arise. The clinical utility of these observations depend on a much larger sample size to identify the relative risk with greater certainty. If our point estimate of relative risk is correct, patients with these variants will have about an 8% risk of schizophrenia which may be of clinical utility in certain families that have experienced the utter devastation that this disease can bring. The clinical utility increases as the relative risk increases. Our data are consistent with a higher relative risk if the miRNA gene with the ultra-rare variant found in the control were found to be involved in diseases other than schizophrenia. In addition, as gene interactions become apparent, relative risk calculations may be refined. Finally, chemically synthesized miRNA analogs with stability within cells may have preventative or therapeutic implications for affected families [37]–[39].

## Supporting Information

Table S1 (0.06 MB XLS)

Table S2 (0.03 MB XLS)

Table S3 (0.05 MB XLS)

Table S4 (0.02 MB XLS)

Table S5 (0.04 MB DOC)

Figure S1 In all pictures: the mature sequences in the stem-loop structure are in uppercase except SNPs in mature sequence are in lower case; sequences outside the mature sequences are in lower case except SNPs outside the mature sequences are in uppercase. Fig. S1a: Function test of miR-510-T/C Variant miR-510-T/C has a ‘T’ (U) to ‘C’ transition in the seed of the predicted miR-510* (3p). Transfection assays show processing of the miR-510-3p product and its ability to knockdown the corresponding ‘si’ target sequence (bar# 8). The ‘T’ (U)/‘C’ mutation produces a pre-miR-510 with much less activity (Bar # 2 vs3, bar #5 vs 6 and bar #8 vs 9). This mutation most likely affects the structure of the pre-miR-510, as it affects the function of mature miR-510 on both strands. Sic-[target]-Si and Sic-[target]-Mi: Dual reporters containing the miRNA target sequences (Si, fully complementary; Mi, partially complementary) in the 3′UTR of the Renilla luciferase gene (for details, see Materials and Methods). fU1-miR-[miRNA] and fU1-miR-[miRNA]-m: miRNA expression vectors containing the primary sequence of a specific miRNA gene (wild type and mutant, respectively) (for details, see Materials and Methods). fU1-miR: Expression vector alone without the miRNA gene inserted. Fig. S1b: Northern blot test of miR-510-T/C Northern blot analyses confirmed that the production of both pre-miR-510 and miR-510-5p/3p were reduced. Top is the result of the blot that was hybridized with miR-510 3p probe; middle is the result that the blot was hybridized with 5p probe; bottom is the result that the blot was hybridized with U2 snoRNA probe and spike-in siRNA probe. U2 was used as RNA sample loading control. SiRNA-1 that target HIV Tat/Rev was used as transfection control. Lanes 1, 2, and 3 are miR-510; Lanes 4 and 5 are transfected with the variant.
(0.49 MB DOC)

Figure S2 Function test of miR-660-C/T Variant miR-660 has a ‘C’ to ‘T’ (U) transition at the 15th position of the mature miRNA. The functional assay data shows it has little effect on the processing of the miRNA. The base change could affect the natural targeting functions of the miRNA since this position affects the 3′end base pairing of miRNA/mRNA. Sic-[target]-Si and Sic-[target]-Mi: Dual reporters containing the miRNA target sequences (Si, fully complementary; Mi, partially complementary) in the 3′UTR of the Renilla luciferase gene (for details, see Materials and Methods). fU1-miR-[miRNA] and fU1-miR-[miRNA]-m: miRNA expression vectors containing the primary sequence of a specific miRNA gene (wild type and mutant, respectively) (for details, see Materials and Methods). fU1-miR: Expression vector alone without the miRNA gene inserted.
(0.03 MB DOC)

Figure S3 Function test of miR-325-G/A Variant miR-325-G/A occurs on the miR-325-3p strand. At this time, there is no information concerning whether or not miR-325* (3p) is functional. Interestingly, our functional assays with the wild type and mutant variants did not generate knockdowns of the ‘si’ target. This miRNA was originally cloned in a murine system and therefore there is no data demonstrating that the human homologue can be expressed and processed in HEK293 cells. (We also analyzed processing of this miRNA in Hela and NIH 3T3 cells, and in both cases were unable to detect knockdown of the complementary target). Sic-[target]-Si and Sic-[target]-Mi: Dual reporters containing the miRNA target sequences (Si, fully complementary; Mi, partially complementary) in the 3′UTR of the Renilla luciferase gene (for details, see Materials and Methods). fU1-miR-[miRNA] and fU1-miR-[miRNA]-m: miRNA expression vectors containing the primary sequence of a specific miRNA gene (wild type and mutant, respectively) (for details, see Materials and Methods). fU1-miR: Expression vector alone without the miRNA gene inserted.
(0.04 MB DOC)

Figure S4 Function test of miR-505-C/T Variant miR-505/505*-C/T has a ‘C’ to ‘T’ (U) transition at the 8th nt (relative to the 5′ end of the upper strand of the mature miR-505). This variant is distal to both Drosha and Dicer cleavage sites. Functional testing of this mutant revealed little difference when compared with the wild type miRNA for both ‘si’ and ‘mi’ targets. Sic-[target]-Si and Sic-[target]-Mi: Dual reporters containing the miRNA target sequences (Si, fully complementary; Mi, partially complementary) in the 3′UTR of the Renilla luciferase gene (for details, see Materials and Methods). fU1-miR-[miRNA] and fU1-miR-[miRNA]-m: miRNA expression vectors containing the primary sequence of a specific miRNA gene (wild type and mutant, respectively) (for details, see Materials and Methods). fU1-miR: Expression vector alone without the miRNA gene inserted.
(0.05 MB DOC)

Figure S5 Function test of miR-509-3-C/T. This miRNA variant has a ‘C’ to ‘T’ (U) transition at the 13th nucleotide of the mature miRNA. (Fig. S5). Our functional assays show that this mutation has a weak effect on the processing of the mature miRNA. This base change could affect the regulation of endogenous targets as the 13th position is important for 3′end base pairing of miRNAs and mRNAs. Sic-[target]-Si and Sic-[target]-Mi: Dual reporters containing the miRNA target sequences (Si, fully complementary; Mi, partially complementary) in the 3′UTR of the Renilla luciferase gene (for details, see Materials and Methods). fU1-miR-[miRNA] and fU1-miR-[miRNA]-m: miRNA expression vectors containing the primary sequence of a specific miRNA gene (wild type and mutant, respectively) (for details, see Materials and Methods). fU1-miR: Expression vector alone without the miRNA gene inserted.
(0.04 MB DOC)
