# Supplementary material for: Evidence for X-Chromosomal Schizophrenia Associated with microRNA Alterations
Source: PLoS One. 2009 Jul 1;4(7):e6121. doi: 10.1371/journal.pone.0006121 (PMC2699475; doi:10.1371/journal.pone.0006121)
Supplement: Table S5 — (0.04 MB DOC) [file pone.0006121.s005.doc]

Table S5. Target genes of miRNAs in which we found ultra-rare cohort-specific variants.

| **Genes** | **Function** | **miRNAs with ultra rare variants have binding site in 3’UTR** |
| --- | --- | --- |
| CLCN5 | Chloride channel 5 (nephrolithiasis 2, X-linked, Dent disease)  Mutations in this gene have been found in Dent disease and renal tubular disorders complicated by nephrolithiasis | Let-7f, miR-502, miR-18b, miR-660, |
| HMGA2 | HMG proteins function as architectural factors and are essential components of the enhancesome. Identification of the deletion, amplification, and rearrangement of this gene that are associated with myxoid liposarcoma suggests a role in adipogenesis and mesenchymal differentiation. | Let-7f, miR-505 |
| NRXN3 | Neurexins are a family of proteins that function in the vertebrate nervous system as cell adhesion molecules and receptors. | Let-7f |
| DISC1 | Disrupted in schizophrenia 1: The protein is involved in neurite outgrowth and cortical development through its interaction with other proteins. This gene is disrupted by a t(1;11)(q42.1;q14.3) translocation which segregates with schizophrenia and related psychiatric disorders in a large Scottish family. | Let-7f, miR-18b, miR-510, miR-188, miR-502 |
| NRG1 | Neuregulin 1: Interacts with the NEU/ERBB2 receptor tyrosine kinase to increase its phosphorylation on tyrosine residues. It induces the growth and differentialtion of epithelial, neuronal, glial and other types of cells. | miR-505 |
| MECP2 | Methyl CpG binding protein 2: Mutations of MECP2 are the cause of some cases of Rett syndrome, a progressive neurologic developmental disorder, and are one of the most common causes of mental retardation in females. | Let-7f, miR-188 miR-325, miR-18b |
| RGS4 | Regulator of G-protein signaling 4: It negatively regulates signaling upstream or at the level of the heterotrimeric G protein and is localized in the cytoplasm. | miR-18b, miR-502 |
| GRM3 | Glutamate receptor, metabotropic 3: L-glutamate is the major excitatory neurotransmitter in the central nervous system and activates both ionotropic and metabotropic glutamate receptors. Glutamatergic neurotransmission is involved in most aspects of normal brain function and can be perturbed in many neuropathologic conditions. | miR-325 |
| ERBB4 | This protein binds to and is activated by neuregulins and other factors and induces a variety of cellular responses including mitogenesis and differentiation. | miR-188, miR-325, miR-502, miR-660 |
| MAGI2 | The protein encoded by this gene interacts with atrophin-1. Atrophin-1 contains a polyglutamine repeat, expansion of which is responsible for dentatorubral and pallidoluysian atrophy. | miR-510 |
| DLG2 | It forms a heterodimer with a related family member that may interact at postsynaptic sites to form a multimeric scaffold for the clustering of receptors, ion channels, and associated signaling proteins. | miR-325, miR-502, miR-510 |
