# Supplementary material for: Evidence for X-Chromosomal Schizophrenia Associated with microRNA Alterations
Source: PLoS One. 2009 Jul 1;4(7):e6121. doi: 10.1371/journal.pone.0006121 (PMC2699475; doi:10.1371/journal.pone.0006121)
Supplement: Figure S1 — In all pictures: the mature sequences in the stem-loop structure are in uppercase except SNPs in mature sequence are in lower case; sequences outside the mature sequences are in lower case except SNPs outside the mature sequences are in uppercase. Fig. S1a: Function test of miR-510-T/C Variant miR-510-T/C has a ‘T’ (U) to ‘C’ transition in the seed of the predicted miR-510* (3p). Transfection assays show processing of the miR-510-3p product and its ability to knockdown the corresponding ‘si’ target sequence (bar# 8). The ‘T’ (U)/‘C’ mutation produces a pre-miR-510 with much less activity (Bar # 2 vs3, bar #5 vs 6 and bar #8 vs 9). This mutation most likely affects the structure of the pre-miR-510, as it affects the function of mature miR-510 on both strands. Sic-[target]-Si and Sic-[target]-Mi: Dual reporters containing the miRNA target sequences (Si, fully complementary; Mi, partially complementary) in the 3′UTR of the Renilla luciferase gene (for details, see Materials and Methods). fU1-miR-[miRNA] and fU1-miR-[miRNA]-m: miRNA expression vectors containing the primary sequence of a specific miRNA gene (wild type and mutant, respectively) (for details, see Materials and Methods). fU1-miR: Expression vector alone without the miRNA gene inserted. Fig. S1b: Northern blot test of miR-510-T/C Northern blot analyses confirmed that the production of both pre-miR-510 and miR-510-5p/3p were reduced. Top is the result of the blot that was hybridized with miR-510 3p probe; middle is the result that the blot was hybridized with 5p probe; bottom is the result that the blot was hybridized with U2 snoRNA probe and spike-in siRNA probe. U2 was used as RNA sample loading control. SiRNA-1 that target HIV Tat/Rev was used as transfection control. Lanes 1, 2, and 3 are miR-510; Lanes 4 and 5 are transfected with the variant. (0.49 MB DOC) [file pone.0006121.s006.doc]

In all pictures: the mature sequences in the stem-loop structure are in uppercase except SNPs in mature sequence are in lower case; sequences outside the mature sequences are in lower case except SNPs outside the mature sequences are in uppercase.

Fig. S1a: Function test of miR-510-T/C

Variant miR-510-T/C has a ‘T’ (U) to ‘C’ transition in the seed of the predicted miR-510* (3p). Transfection assays show processing of the miR-510-3p product and its ability to knockdown the corresponding ‘si’ target sequence (bar# 8). The ‘T’ (U)/’C’ mutation produces a pre-miR-510 with much less activity (Bar # 2 vs3, bar #5 vs 6 and bar #8 vs 9). This mutation most likely affects the structure of the pre-miR-510, as it affects the function of mature miR-510 on both strands.

Sic-[target]-Si and Sic-[target]-Mi: Dual reporters containing the miRNA target sequences (Si, fully complementary; Mi, partially complementary) in the 3’UTR of the Renilla luciferase gene (for details, see Materials and Methods).

fU1-miR-[miRNA] and fU1-miR-[miRNA]-m: miRNA expression vectors containing the primary sequence of a specific miRNA gene (wild type and mutant, respectively) (for details, see Materials and Methods).

fU1-miR: Expression vector alone without the miRNA gene inserted.

Fig. S1b: Northern blot test of miR-510-T/C

Northern blot analyses confirmed that the production of both pre-miR-510 and miR-510-5p/3p were reduced. Top is the result of the blot that was hybridized with miR-510 3p probe; middle is the result that the blot was hybridized with 5p probe; bottom is the result that the blot was hybridized with U2 snoRNA probe and spike-in siRNA probe. U2 was used as RNA sample loading control. SiRNA-1 that target HIV Tat/Rev was used as transfection control. Lanes 1, 2, and 3 are miR-510; Lanes 4 and 5 are transfected with the variant.
