# Supplementary material for: Evidence for X-Chromosomal Schizophrenia Associated with microRNA Alterations
Source: PLoS One. 2009 Jul 1;4(7):e6121. doi: 10.1371/journal.pone.0006121 (PMC2699475; doi:10.1371/journal.pone.0006121)
Supplement: Figure S2 — Function test of miR-660-C/T Variant miR-660 has a ‘C’ to ‘T’ (U) transition at the 15th position of the mature miRNA. The functional assay data shows it has little effect on the processing of the miRNA. The base change could affect the natural targeting functions of the miRNA since this position affects the 3′end base pairing of miRNA/mRNA. Sic-[target]-Si and Sic-[target]-Mi: Dual reporters containing the miRNA target sequences (Si, fully complementary; Mi, partially complementary) in the 3′UTR of the Renilla luciferase gene (for details, see Materials and Methods). fU1-miR-[miRNA] and fU1-miR-[miRNA]-m: miRNA expression vectors containing the primary sequence of a specific miRNA gene (wild type and mutant, respectively) (for details, see Materials and Methods). fU1-miR: Expression vector alone without the miRNA gene inserted. (0.03 MB DOC) [file pone.0006121.s007.doc]

In all pictures: the mature sequences in the stem-loop structure are in uppercase except SNPs in mature sequence are in lower case; sequences outside the mature sequences are in lower case except SNPs outside the mature sequences are in uppercase.

Fig. S2: Function test of miR-660-C/T

Variant miR-660 has a ‘C’ to ‘T’ (U) transition at the 15^th^ position of the mature miRNA. The functional assay data shows it has little effect on the processing of the miRNA. The base change could affect the natural targeting functions of the miRNA since this position affects the 3’end base pairing of miRNA/mRNA.

Sic-[target]-Si and Sic-[target]-Mi: Dual reporters containing the miRNA target sequences (Si, fully complementary; Mi, partially complementary) in the 3’UTR of the Renilla luciferase gene (for details, see Materials and Methods).

fU1-miR-[miRNA] and fU1-miR-[miRNA]-m: miRNA expression vectors containing the primary sequence of a specific miRNA gene (wild type and mutant, respectively) (for details, see Materials and Methods).

fU1-miR: Expression vector alone without the miRNA gene inserted.
