# Supplementary material for: Evidence for X-Chromosomal Schizophrenia Associated with microRNA Alterations
Source: PLoS One. 2009 Jul 1;4(7):e6121. doi: 10.1371/journal.pone.0006121 (PMC2699475; doi:10.1371/journal.pone.0006121)
Supplement: Figure S3 — Function test of miR-325-G/A Variant miR-325-G/A occurs on the miR-325-3p strand. At this time, there is no information concerning whether or not miR-325* (3p) is functional. Interestingly, our functional assays with the wild type and mutant variants did not generate knockdowns of the ‘si’ target. This miRNA was originally cloned in a murine system and therefore there is no data demonstrating that the human homologue can be expressed and processed in HEK293 cells. (We also analyzed processing of this miRNA in Hela and NIH 3T3 cells, and in both cases were unable to detect knockdown of the complementary target). Sic-[target]-Si and Sic-[target]-Mi: Dual reporters containing the miRNA target sequences (Si, fully complementary; Mi, partially complementary) in the 3′UTR of the Renilla luciferase gene (for details, see Materials and Methods). fU1-miR-[miRNA] and fU1-miR-[miRNA]-m: miRNA expression vectors containing the primary sequence of a specific miRNA gene (wild type and mutant, respectively) (for details, see Materials and Methods). fU1-miR: Expression vector alone without the miRNA gene inserted. (0.04 MB DOC) [file pone.0006121.s008.doc]

In all pictures: the mature sequences in the stem-loop structure are in uppercase except SNPs in mature sequence are in lower case; sequences outside the mature sequences are in lower case except SNPs outside the mature sequences are in uppercase.

Fig. S3: Function test of miR-325-G/A

Variant miR-325-G/A occurs on the miR-325-3p strand. At this time, there is no information concerning whether or not miR-325* (3p) is functional. Interestingly, our functional assays with the wild type and mutant variants did not generate knockdowns of the ‘si’ target. This miRNA was originally cloned in a murine system and therefore there is no data demonstrating that the human homologue can be expressed and processed in HEK293 cells (We also analyzed processing of this miRNA in Hela and NIH 3T3 cells, and in both cases were unable to detect knockdown of the complementary target).

Sic-[target]-Si and Sic-[target]-Mi: Dual reporters containing the miRNA target sequences (Si, fully complementary; Mi, partially complementary) in the 3’UTR of the Renilla luciferase gene (for details, see Materials and Methods).

fU1-miR-[miRNA] and fU1-miR-[miRNA]-m: miRNA expression vectors containing the primary sequence of a specific miRNA gene (wild type and mutant, respectively) (for details, see Materials and Methods).

fU1-miR: Expression vector alone without the miRNA gene inserted.
