# Supplementary material for: Evidence for X-Chromosomal Schizophrenia Associated with microRNA Alterations
Source: PLoS One. 2009 Jul 1;4(7):e6121. doi: 10.1371/journal.pone.0006121 (PMC2699475; doi:10.1371/journal.pone.0006121)
Supplement: Figure S4 — Function test of miR-505-C/T Variant miR-505/505*-C/T has a ‘C’ to ‘T’ (U) transition at the 8th nt (relative to the 5′ end of the upper strand of the mature miR-505). This variant is distal to both Drosha and Dicer cleavage sites. Functional testing of this mutant revealed little difference when compared with the wild type miRNA for both ‘si’ and ‘mi’ targets. Sic-[target]-Si and Sic-[target]-Mi: Dual reporters containing the miRNA target sequences (Si, fully complementary; Mi, partially complementary) in the 3′UTR of the Renilla luciferase gene (for details, see Materials and Methods). fU1-miR-[miRNA] and fU1-miR-[miRNA]-m: miRNA expression vectors containing the primary sequence of a specific miRNA gene (wild type and mutant, respectively) (for details, see Materials and Methods). fU1-miR: Expression vector alone without the miRNA gene inserted. (0.05 MB DOC) [file pone.0006121.s009.doc]

In all pictures: the mature sequences in the stem-loop structure are in uppercase except SNPs in mature sequence are in lower case; sequences outside the mature sequences are in lower case except SNPs outside the mature sequences are in uppercase.

Fig. S4: Function test of miR-505-C/T

Variant miR-505/505*-C/T has a ‘C’ to ‘T’ (U) transition at the 8^th^ nt (relative to the 5’ end of the upper strand of the mature miR-505). This variant is distal to both Drosha and Dicer cleavage sites. Functional testing of this mutant revealed little difference when compared with the wild type miRNA for both ‘si’ and ‘mi’ targets.

Sic-[target]-Si and Sic-[target]-Mi: Dual reporters containing the miRNA target sequences (Si, fully complementary; Mi, partially complementary) in the 3’UTR of the Renilla luciferase gene (for details, see Materials and Methods).

fU1-miR-[miRNA] and fU1-miR-[miRNA]-m: miRNA expression vectors containing the primary sequence of a specific miRNA gene (wild type and mutant, respectively) (for details, see Materials and Methods).

fU1-miR: Expression vector alone without the miRNA gene inserted.
