# Supplementary material for: Evidence for X-Chromosomal Schizophrenia Associated with microRNA Alterations
Source: PLoS One. 2009 Jul 1;4(7):e6121. doi: 10.1371/journal.pone.0006121 (PMC2699475; doi:10.1371/journal.pone.0006121)
Supplement: Figure S5 — Function test of miR-509-3-C/T. This miRNA variant has a ‘C’ to ‘T’ (U) transition at the 13th nucleotide of the mature miRNA. (Fig. S5). Our functional assays show that this mutation has a weak effect on the processing of the mature miRNA. This base change could affect the regulation of endogenous targets as the 13th position is important for 3′end base pairing of miRNAs and mRNAs. Sic-[target]-Si and Sic-[target]-Mi: Dual reporters containing the miRNA target sequences (Si, fully complementary; Mi, partially complementary) in the 3′UTR of the Renilla luciferase gene (for details, see Materials and Methods). fU1-miR-[miRNA] and fU1-miR-[miRNA]-m: miRNA expression vectors containing the primary sequence of a specific miRNA gene (wild type and mutant, respectively) (for details, see Materials and Methods). fU1-miR: Expression vector alone without the miRNA gene inserted. (0.04 MB DOC) [file pone.0006121.s010.doc]

In all pictures: the mature sequences in the stem-loop structure are in uppercase except SNPs in mature sequence are in lower case; sequences outside the mature sequences are in lower case except SNPs outside the mature sequences are in uppercase.

Fig. S5: Function test of miR-509-3-C/T

This miRNA variant has a ‘C’ to ‘T’ (U) transition at the 13^th^ nucleotide of the mature miRNA. (Fig.S5). Our functional assays show that this mutation has a weak effect on the processing of the mature miRNA. This base change could affect the regulation of endogenous targets as the 13^th^ position is important for 3’end base pairing of miRNAs and mRNAs.

Sic-[target]-Si and Sic-[target]-Mi: Dual reporters containing the miRNA target sequences (Si, fully complementary; Mi, partially complementary) in the 3’UTR of the Renilla luciferase gene (for details, see Materials and Methods).

fU1-miR-[miRNA] and fU1-miR-[miRNA]-m: miRNA expression vectors containing the primary sequence of a specific miRNA gene (wild type and mutant, respectively) (for details, see Materials and Methods).

fU1-miR: Expression vector alone without the miRNA gene inserted.
